# Clinical characteristics, diagnosis, treatment, and outcome of patients with liver abscess due to *Aspergillus spp:* a systematic review of published cases

**DOI:** 10.1186/s12879-024-09226-y

**Published:** 2024-03-22

**Authors:** Igor Dumic, Enzo Marasco Caetano, Sidney Marcel Domingues, Ivana Pantic, Milan Radovanovic, Libardo Rueda Prada, Charles W Nordstrom, Marina Antic, Tamara Milovanovic, Magdalena Kotseva, Amteshwar Singh, Shweta FNU

**Affiliations:** 1 Mayo Clinic College of Medicine and Science, Rochester, MN USA; 2 Department of Hospital Medicine, Mayo Clinic Health System, Eau Claire, WI USA; 3 Municipal University of São Caetano do Sul, Sao Paulo, Brazil; 4 Clinic for Gastroenterology and Hepatology, University Clinical Center of Serbia, Belgrade, Serbia; 5 Department of Hospital Medicine, Mayo Clinic Jacksonville, Florida, USA; 6 Faculty of Medicine, University of Belgrade, Belgrade, Serbia; 7 Internal Medicine Residency Program, Franciscan Health, Olympia Fields, IL USA; 8 Department of Medicine, Johns Hopkins University, Baltimore, MD USA; 9 Department of Infectious Diseases, Mayo Clinic Health System, Eau Claire, WI USA

**Keywords:** Liver abscess, Aspergillus, Immunosupression

## Abstract

**Background:**

*Aspergillus spp* liver abscess is a relatively rare entity and thus far no systematic review has been performed examining patients’ demographics, clinical manifestations, diagnosis, management, and outcome.

**Methods:**

We performed a systematic review of the literature using MEDLINE and LILACS databases. We searched for articles published in the period from January 1990 to December 24, 2022, to identify patients who developed liver abscesses due to *Aspergillus* spp.

**Results:**

Our search yielded 21 patients all of whom had invasive aspergillosis confirmed on liver biopsy. Of these patients 81% were adults, and 60% were males. The majority (86%) of patients were immunocompromised and 95% had symptomatic disease at the time of diagnosis. The most common symptoms were fever (79%), abdominal pain (47%), and constitutional symptoms (weight loss, chills, night sweats, fatigue) (38%). Liver enzymes were elevated in 50%, serum galactomannan was positive in 57%, and fungal blood cultures were positive in only 11%. Co-infection with other pathogens preceded development of apsergillosis in one-third of patients, and the majority of the abscesses (43%) were cryptogenic. In the remaining patients with known source, 28% of patients developed liver abscess through dissemination from the lungs, 19% through the portal vein system, and in 10% liver abscess developed through contiguous spread. The most common imaging modality was abdominal computerized tomography done in 86% of patients. Solitary abscess was present in 52% of patients while 48% had multiple abscesses. Inadequate initial empiric therapy was prescribed in 60% of patients and in 44% of patients definite treatment included combination therapy with two or more antifungal agents. Percutaneous drainage of the abscesses was done in 40% of patients, while 20% required liver resection for the treatment of the abscess. Overall mortality was very high at 38%.

**Conclusion:**

Further studies are urgently needed for a better understanding of pathophysiology of liver aspergillosis and for developement of newer blood markers in order to expedite diagnosis and decrease mortality.

## Background

A liver abscess (LA) is a rare condition characterized by the formation of a purulent cavity by microorganisms in the liver [1–5]. LA is classified as bacterial, protozoan (amoebic), or fungal [1–5]. The most common causes of bacterial (pyogenic) liver abscesses (BLA) are *Escherichia coli*, *Klebsiella spp*, *Streptococcus anginosus group, Staphylococcus aureus*, and anaerobes. Amebic liver abscesses (ALA) are the most common manifestation of extra-intestinal amebiasis caused by *Entamoeba histolytica*.Fungal pathogens, however, are comparitively rare cause of LA [1–5].

The incidence of BLA varies by geography. For example, in the North American population the incidence is around 2 per 100,000, but 17.6 per 100,000 inhabitants in Taiwan. Meanwhile in Sweden, the incidence of BLA has increased almost three fold over the last decade, from 1.8/100,000 person-years in 2011 to 5.2/100,000 person-years in 2020 which is partially explained by their aging population [6].

Fungal infections in the liver are less common compared to BLA. *Candida spp* and *Aspergillus spp* are the most common causative agents in patients with hematological malignancy [7–12]. The incidence of these infections has decreased due to the use of antifungal prophylaxis, particularly amongst patients with cancer on immunosuppressive therapy. The most common *Aspergillus spp* causing infections in humans are: *A. fumigatus*, *A. flavus*, *A. niger, and A. terreus* [7, 8]. The most common and most severe form of aspergillosis is invasive aspergillosis (IA), followed by a chronic, allergic form termed chronic bronchopulmonary aspergillosis [9–11, 13]. While the lungs and upper respiratory organs are most commonly affected in aspergillosis, other visceral organs are typcially infected following dissemination from the primary lung focus [9, 10].

*Aspergillus spp* liver abscess or invasive liver aspergillosis (ILA) is a rare extrapulmonary manifestation of IA [14]. In a recent autopsy study, the liver was amongst the least commonly affected extrapulmonary sites, and hepatic aspergillosis was found in only 10% of patients with fatal IA compared to central nervous system and cardiac IA that were the most common extrapulmonary sites involved, in 24% of cases each [15]. There is a paucity of evidence about patients’ characteristics, risk factors, management, and outcomes of patients with ILA. The data we have about this rare clinical manifestation of IA are based on case reports, case series, and expert opinions. Due to the rarity of the disease, there are no retrospective or prospective studies on this specific topic. Therefore, we performed a scoping review of case reports and case series to understand more about ILA and discuss our findings in the context of established knowledge on the more common etiologies of liver abscesses(i.e.,BLA and ALA.)

## Methods

A scoping review of the literature was performed by searching the MEDLINE database via PubMed search engine and Latin American and Caribbean Health Sciences Literature (LILACS) database via Bvsalud search engine from January 1990 to December 24, 2022, in order to identify patients who developed liver abscess due to *Aspergillus spp* using the Preferred Reporting Items for Systematic Reviews and Meta-Analyses (PRISMA) extension for scoping review (ScR) methodology. The keywords used for the literature search were: “aspergillus or aspergillosis”, “liver or hepatic”, and “infection or abscess”. Furthermore, the reference list of identified articles was manually screened to identify additional cases that can be included in our analysis.

Two authors (I.D. and E.M.) independently and blindly screened the titles, abstracts, and full manuscripts of the identified articles reporting cases of microbiologically proven ILA. Articles that were not case reports and did not report aspergillosis, articles written in a language other than English and Portuguese, and articles that did not contain sufficient information were excluded. Any discrepancies or uncertainties were resolved by the first author (I.D.).

**Fig. 1:**
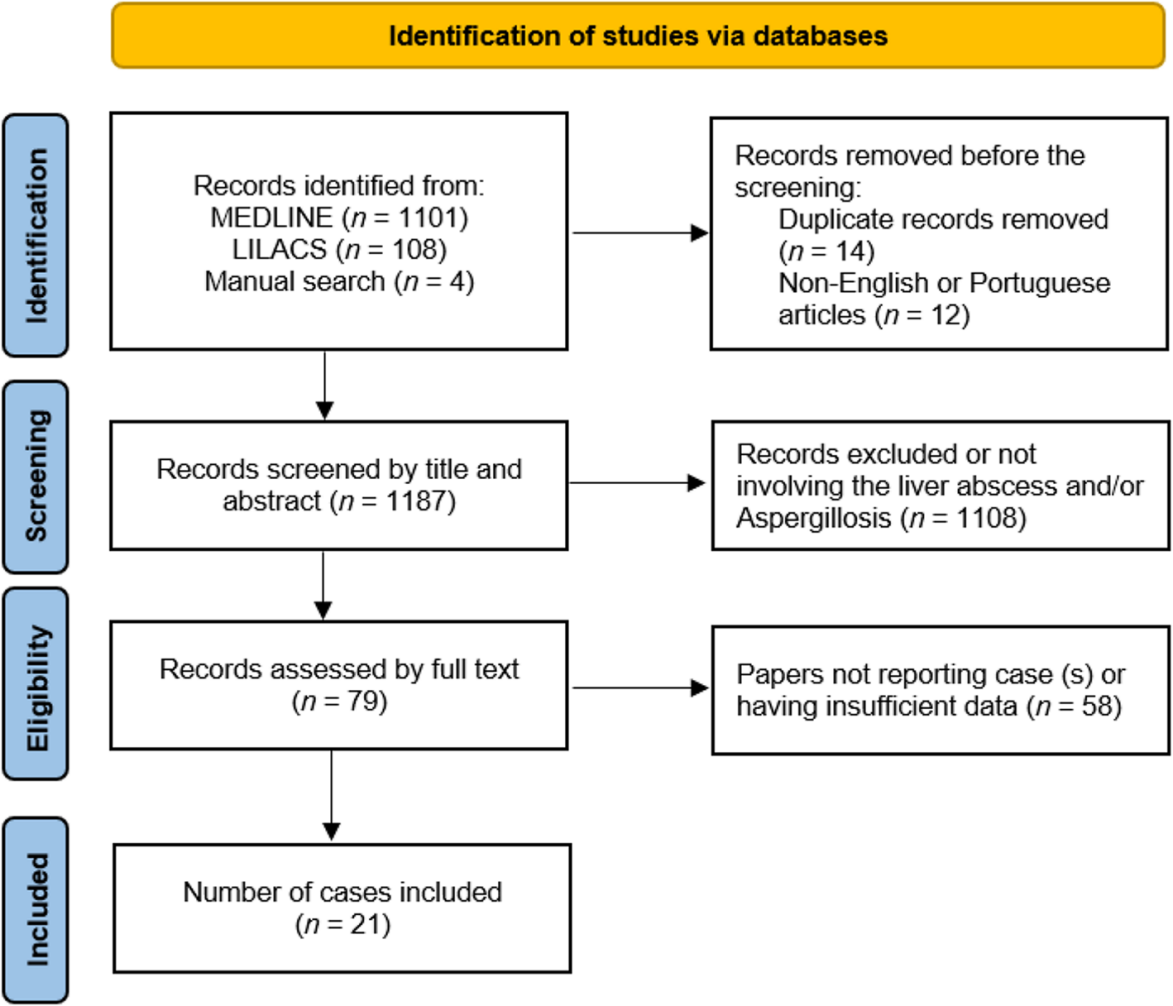
Prisma flowchart detailing the search results

A total of 1213 articles were identified by the initial search, out of which 14 were duplicates.. The selection process resulted in a total of 21 articles in this study [16–35]. A detailed PRISMA flowchart is illustrated in Fig. 1.

After the final articles were selected, data was collected and the following variables were extracted: age, gender, comorbidities/immunosuppression (high dose steroids, concurrent or recent chemotherapy in the past 1 year, solid organ transplant [SOT] or hematopoietic stem cell transplant [HSCT], hematologic malignancy - not achieved remission, Acquired Immunodeficiency Syndrome [AIDS], use of Tumor Necrosis Factor [TNF] alpha or anti-CD20 therapy in past 2 years, diabetes mellitus [DM]), presence of any other infections, primary Aspergillus focus and portal of entry, symptoms of abdominal pain, fever, or constitutional symptoms (weight loss, chills, night sweats, fatigue), laboratory findings (liver function tests [LFTs], serum galactomannan [GM], liver biopsy, cultures), imaging, management, and outcome.

All cases included met criteria for definite IA by liver biopsy specimen either demonstrating fungal hyphae and/or fungal cultures growing *Aspergillus* spp. [10, 36].

## Results

### Demographic characteristics

The total number of cases identified in this scoping review was 21 patients, out of which 17 were adults. The mean age was 38 ± 19 years. Out of 4 pediatric cases, two were males and one was female, while sex was not reported in one child. Immunosuppression was present in 86% of these patients; most commonly due to chemotherapy and hematological malignancy – not achieved remission (35%), followed by organ transplants (20%), while other immune-compromising conditions such as AIDS, aplastic anemia, and splenectomy were less common. For the pediatric group, 3 out of 4 had inherited immunodeficiencies **(**Table 1**).**

**Table 1:** Summarize demohraphic information, immune status, presumable port of entry, management and outcome of patients with liver abscess due to aspergilus spp.

Demographic Characteristics (*n* = 21)	*n* (%)
Gender	
Male	12 (57.1%)
Female	8 (38.1%)
Not reported	1 (4.8%)
Age (years)	
≤ 18	5 (23.8%)
19–64	14 (66.7%)
≥ 65	2 (9.5%)
**Immune Status**	
Immunosuppressed	18 (85.7%)
Aplastic anemia	4 (22.2%)
Inherited immune deficiencies	3 (16.7%)
Lymphoma/Leukemia	3 (16.7%)
Organ transplant	3 (16.7%)
AIDS	2 (11.1%)
Immunocompetent	3 (14.3%)
**Presumed Port of Entry**	
Cryptogenic (unknown)	9 (42.9%)
Hematogenous spread from the lungs	6 (28.6%)
Portal entry from GI tract	4 (19.0%)
Contiguous Spread	2 (9.5%)
**Imaging**	
CT scan of the abdomen	17 (80.9%)
Single abscess	10 (58.8%)
Multiple abscesses	7 (41.2%)
Ultrasound	9 (42.9%)
MRI	1 (4.8%)
**Empiric therapy**	
Reported	20 (95.24%)
Inadequate	12 (60.0%)
Adequate	8 (40.0%)
**Definite Therapy**	
Combination (Medical + Procedure)	11 (52.4%)
Medical management only	8 (38.1%)
Procedure management only	2 (9.5%)
**Outcome**	
Recovered	13 (61.9%)
Death	8 (38.1%)

*AIDS* Acquired Immune Deficiency Syndrome: *GI* gastrointestinal: *CT* Computerized Tomography: *MRI* Magnetic Resonance Imaging

### Source of infection and coinfection

The abscesses were cryptogenic in 43% of cases. A presumed pulmonary source of infection with secondary liver involvement by hematogenous dissemination was documented in 28% of cases. Liver infection by dissemination from the gastrointestinal tract via the portal vein system was documented in in 19%. In the remaining 10%, spread to liver occured contiguously through the skin from right-sided rib osteomyelitis and from partially treated left adrenal gland aspergillosis. Co-infection with another pathogen was present in 33% of cases.

### Clinical presentation

Symptomatic disease was present in 95% of cases, while in a single case (5%), the disease was asymptomatic and discovered by serial monitoring of galactomannan test in a post-HSCT patient. The most commonly reported symptoms included fever, abdominal pain, and constitutional symptoms in 79%, 47%, and 38% of patients, respectively **(**Table 1**).**

### Laboratory findings

All 21 patients had a liver biopsy with *Aspergillus spp* demonstrated from the liver tissue that met the criteria for confirmed invasive aspergillosis. There was a high variability of reported laboratory findings and many case reports had not reported their full spectrum of laboratory investigation. Furthermore, as many patients were on chemotherapy or in the post-transplant period, complete blood cell counts were frequently affected by the primary problem. LFTs were reported in 14 cases and of those, 50% cases reported liver enzyme elevation. Serum GM and blood cultures were not reported in many cases. In those who reported it, serum GM was positive in 57%. Fungal blood cultures for *Aspergillus* were positive in only 1 of 9 patients (positivity rate of 11%).

### Imaging

The most common imaging modality was abdominal computerized tomography (CT) performed in 81% of patients at any point during the management.Abdominal ultrasound (US), as an initial test, was performed in 42% of patients. Abdominal magnetic resonance imaging (MRI) was done in only one patient. Abscesses were solitary in 52% of patients, while multiple abscesses were reported in 48%. The abscess size was variable and ranged from 2.2 cm to 9 cm in diameter.

### Management

Initial empiric antimicrobial treatment was inadequate in 60% of patients where a bacterial pathogen was suspected and Aspergillus-specific antimicrobial treatment was given only after the cultures were reported. In 53% of patients, monotherapy with either amphotericin, itraconazole, voriconazole, or caspofungin was utilized, while in the remaining 47% combination therapy with any of the two agents was used.

### Outcome

Overall mortality was substantial and death due to complications from overwhelming infection or related complications occurred in 8 out of 21 patients (38%), prior to the diagnosis being established or during the treatment course.

## Discussion

### Demographics, comorbidities and the risk factors

Similar to BLA and ALA, ILA occurred more frequently in men. It remains unclear why liver abscesses are more common in men, but across the studies that seems to be a consistent finding regardless of the etiology of liver abscess [2, 4, 5]. In patients with ALA, this noticeable discrepancy could be explained by several mechanisms, including the effect of testosterone, and by alcohol consumption which is traditionally more prominent in men. In cases of *E. histolytica* infection, alcohol consumption could contribute to higher infection rates since it has been hypothesized that locally produced alcoholic drinks in endemic regions (e.g., palm tree wine) can contain a significant amount of *E. histolytica* [2, 37]. 

A recent study from Sweden demonstrated that BLA incidence has increased over the last decade, and this was attributed to their aging population [6]. Older age is considered a risk factor for BLA but not for ILA. In our review, the mean age was 38 years which is much younger than 65 years as reported in previous studies on BLA, yet similar to the mean age of patients with ALA of 41 years [2, 4, 5, 38]. The reason why ILA is a disease of a younger population is probably related to the predispostion of lymphoma and leukemia for this popluation, and organ transplant recipients tend to be younger.

The risk factors for BLA, ALA, and ILA are vastly different **(**Table 2**).** The most recognized risk factors for the development of BLA are advanced age, uncontrolled DM, liver trauma, intraabdominal surgery, use of proton pump inhibitors (PPI), and biliary pathology [2, 4, 5, 39]. The most important risk factors for amebic liver abscesses (ALA) are poor sanitation, travel to endemic areas, and malnutrition [2] . In contrast, patients with fungal liver abscess are usually profoundly immunocompromised as evidenced by these review findings. The risk factor for hepatosplenic candidiasis, including liver abscess, is prolonged and severe neutropenia [40]. Only 3 patients in our review were immunocompetent. In these patients, aspergillosis developed during complications of postpartum necrotizing fascitis [20],as a consequence of gastric ulcer perforation which communicated with the left liver lobe [28], or from contiguous spread from adrenal gland aspergillosis [41]. All other patients (86%) were in an immunocompromised state: either HSCT or SOT recipients, or patients who were undergoing high intensity chemotherapy for hematologic malignancies (leukemia and lymphoma). Prolonged neutropenia is a strong risk factor for *Aspergillus spp* infection in patients with hematologic malignancies [42]. Children who developed ILA typically had a profound inherited immunodeficiency in the form of chronic granulomatous disease (CGD), purine nucleoside phosphorylase deficiency (PNP), or common variable immunodeficiency (CVID) [22, 23, 29].

**Table 2:** This table illustrates differences among bacterial, amebic and Aspergillus liver abscesses

	Bacterial	Amebic	Aspergillus
Age	Older age	Middle age	Younger age
Gender	Male predominant	Male predominant (7–10 times more common in adult men)	Male predominant
Main risk factors	• Older age • Diabetes • Malnutrition	• Poor sanitation • Travel to endemic area • Oral and anal sex • Alcohol use	• Immunosuppression • Malignancy especially hematological e.g., ALL, AML • Organ transplant/bone marrow transplant
Most common pathogens	*Escherichia coli, Klebsiella spp, Streptococcus anginosus group, Staphylococcus aureus*, and anaerobes	*Entamoeba histolytica*	*Aspergillus spp *
Initial source of infection	• Biliary disease (50–60%) • Cryptogenic (35%)	Infection with *E. histolytica* cyst via fecal-oral transmission	• Cryptogenic (43%) • Primary lung infection with hematogenous spread (28%) • Intra-abdominal GI infection (19%)
Clinical manifestations	• Fever • Abdominal pain • Constitutional symptoms • Jaundice • Septic emboli to eye, brain, meninges seen in *Klebsiella* BLA	• Fever • Abdominal pain • Constitutional symptoms • Concurrent diarrhea in < 1/3 of cases • Jaundice	• Fever • Abdominal pain • Constitutional symptoms • Coinfection with other pathogens (33%)
Laboratory findings	• Leucocytosis common • LFTs derangement common • Increase in inflammatory markers (CRP, ESR) • In 30% blood cultures negative	• E. histolytica serology is useful to distinguish amoebic from BLA in non endemic regions • Serology can be negative in the 1st week of infection	• Galactomannan positive 57% • Blood cultures positive 11%
Imaging characteristics	• Usually, multiple lesions in the right lobe of the liver • Abscess size < 10 mm in diameter • Septations are common • Gas within abscess: suspect *Klebsiella spp*	• Typically, solitary lesion • Subcapsular • Abscess size 5–10 cm (depends on the ALA form – chronic indolent abscesses are larger) • Most common location: the posterior part of the right lobe (70–80%)	• Solitary abscess (52%) • Multiple abscesses (48%) • Abscess size 2.2–9 cm
Mortality	• Mortality 2.5–19% • Highest mortality in biliary origin compared to other causes • Higher risk of spontaneous rupture in *Klebsiella* BLA	• Mortality 1–3% • Excellent prognosis as they are very sensitive to antimicrobial medical therapy	• Mortality 38%

Other recently described risk factors for IA include intensive care unit (ICU) stay and preceding bacterial or viral infection [43–45]. In this review, 33% of patients had co-infection with another pathogen around the time of liver aspergillosis diagnosis. These co-infections are likely additional risk factors which contribute to development of IA through epithelial damage, allowing for easier dissemination of aspergillosis. In fact, up to 60% of patients diagnosed with invasive pulmonary aspergillosis in an autopsy series were found to have co-infection with another pathogen [15], however in this review it was 33%.

### Pathogenesis of liver aspergillosis

*Aspergillus* is a ubiquitous environmental fungus. While it is a harmless colonizer for the majority of immunocompetent people, it is an opportunistic pathogen in people with defects in cellular and/or humoral immunity. Most IA involves the lungs with inhalation of spores as the most common portal of entry, whereas the visceral organs are usually affected by hematogenous dissemination. Following the lungs, the most common affected organs are the paranasal sinuses and the brain [11, 13, 46]. The liver is very rarely affected in aspergillosis, although some autopsy reports suggest that gastrointestinal (including liver) aspergillosis might be underreported [17, 47].

Of the 21 patients with ILA described here, 28% had disease due to hematogenous seeding from the infectious foci in the lungs, while in 19% of cases the portal of entry was presumed to be the gastrointestinal tract. A gastrointestinal portal of entry has been hypothesized to occur due to profound neutropenia, mucositis and damage to the intestinal epithelium during chemotherapy which allows the fungus to migrate and seed into the liver [17] or due to disruption of intestinal barries during intraabdominal surgery as illustrated in remaining 3 cases [16, 20, 28]. In these cases patients had abdominal surgery for various reasons (perforated peptic ulcer, necrotizing facitis following Cesarean section, and liver transplant) and developed liver abscess 2–4 weeks after that. Cryptogenic liver abscesses were the most commonly documented in 43% in cases where no portal of entry could be identified. Given its angioinvasive features, it is not surprising that the majority of extrapulmonary aspergillosis results from hematogenous dissemination. In two patients the infection occured through contiguous spread from the rib osteomyelitis and from left adrenal gland aspergilosis [41].

### Clinical characteristics and laboratory analysis

Patients with BLA usually present with fever, leukocytosis, and abdominal pain **(**Table 2**).** Patients with ALA are more likely to present with nausea, diarrhea, and protracted constitutional symptoms [48]. Patients with ILA, however, may present differently. Due to profound immunosuppression, these patients are less likely to mount a leukocytosis [49]. Fever, however, remains the most common, and sometimes the only, sign of the infection.This is particularly true in patients with prolonged and severe neutropenia (absolute neutrophil count of less than 0.5 × 10^9^/L (< 500/μL),). Although less common than in patients with BLA, we found fever to be the most common sign of infection in patients with ILA, occuring in 79%. Abdominal pain was present in 47% of cases and we found constitutional symptoms to be present in 38%. Of note, in some immunocompetent patients the liver can be affected diffusely by a disseminated form of aspergillosis with morphologic features of granuloma rather than a well-formed abscess [50]. Liver enzymes were reported in 50% of the 14 cases that reported this information, and all of these patients had mild to moderate elevation in transaminases with hepatocellular pattern of liver injury. Cholestatic pattern of liver injury was not observed in any of the patients who reported the liver function test and one patient had mixed patter of liver injury.

### Diagnosis

A definite diagnosis of IA is established by demonstrating fungal hyphae in tissue specimens or by demonstrating *Aspergillus* spp. growth in fungal cultures of liver tissue. In this review, we included only case reports and case series that fulfilled this definition. Other laboratory (serum GM, beta D glucan, fungal blood cultures) and imaging findings might assist in establishing diagnosis but are not sufficient [10, 51].

The reported sensitivity of serum GM antigen index can vary from 30 to 100%, and specifity is generally reported as > 75%. These values vary significantly between *Aspergillus* and non-*Aspergillus* fungi, testing assay, and host factors such as age and prior HSCT or SOT. While galactomannan is relased into serum from the cell wall of replicating *Aspergillus* species, it is also present in the cell wall of several other fungi and can cross react with antibiotics such as piperacillin-tazobactam and amoxicillin-clavulanate. A low yield from fungal blood cultures is true for other fungal pathogens too, for example *Candida spp* where sensitivity of blood cultures is limited, and up to 70% of patients with hepatosplenic candidiasis might not have documented fungemia [52, 53].

The findings from this report with regard to the serum GM test and fungal blood cultures must be interpreted with caution since many authors of these case reports had not reported the results of these tests. However, if we take into account only the cases that reported results, a positive serum GM was seen in 57% of patients, and fungal blood cultures were positive in 11%. This is in alignment with reports from the European Society of Clinical Microbiology and Infectious Diseases (ESCMID) [51] which state that the serum GM test is more sensitive than fungal blood culture for the diagnosis of invasive aspergillosis. In one case report from Italy, the diagnosis was challenging when the clinical and radiological findings mimicked hepatosplenic candidiasis. In this instance, the diagnosis was established initially by assessment of *Aspergillus* specific T cells by an enzyme-linked immunospot (ELISPOT) assay that demonstrated a high number of Aspergillus-specific T cells producing interleukin-10 [TH2(IL-10)] and a low number of Aspergillus-specific T cells producing gamma interferon [TH1(IFN-γ)] [33]. In this case, the diagnosis was later confirmed by demonstrating fungal growth in liver biopsy specimen and pathohistology.

While serology can be useful to establish a diagnosis in patients with ALA, for ILA and for BLA serologies do not have reliable diagnostic utility.

### Imaging

For diagnosis of LA, abdominal CT and US are the most commonly utilized diagnostic tools, with high sensitivity (US: 85–95%; CT: 100%) [54]. ALA are greater in size and more commonly solitary compared to BLA which are more frequently multiple and bilobar [55]. In this review of ILA, 52% had a solitary abscess and 48% had multiple abscesses.

### Management

The guidelines of the Infectious Diseases Society of America [10] recommend voriconazole as initial therapy for invasive aspergillosis based on randomized clinical trials that demonstrated voriconazole to be more efficient than amphotericin B deoxycholate in relation to survival and clinical improvement (71% vs 58%) [10]. In the current review, only 3 patients received voriconazole monotherapy, 5 recieved amphotericin monotherapy, and 8 received combination of 2 or more antifungal agents. Of 8 patients who received combination therapy, 5 patients received voriconazole plus echinocandin. Of these 5 patients, 2 died. Combination of voriconazole with echinocandin might provide mortality benefit in certain patient populations [56]. These discrepancies with IDSA guidelines are due to the majority of the older reports being published prior to 2016 when the latest IDSA guidelines on aspergilosis management were published.

Amongst the 21 patients described in this systematic review, surgical resection of the liver abscess was performed in 20%, while percutaneous drainage was adequate for source control in 40%. This highlights the challanges associated with treatment of liver aspergillosis and emphasizes that medical management alone is frequently insufficient. Whether or not percutaneous catheter drainage of liver abscess improves outcomes in patients with ILA is debatable. While it seems intuitive that faster source control by aspiration or surgical drainage would potentially lead to a better outcome, firm data are lacking. Moreover patients with ILA are often too ill to undergo any type of procedure.

### Outcome

The mortality of liver abscesses as a whole has decreased dramatically over the years [2]. This improvement in morbidity and mortality is attributed to better and more available imaging techniques, and a larger armementarium of medication available to treat liver abscesses. In our review, the mortality of patients with ILA was 38% which was much higher compared to ALA and BLA **(**Table 2). This higher mortality is due to inherent risk factors in this patient population who tend to be much sicker, immunocompromised, and more challanging to diagnose. Additionally, in ILA patients, the diagnosis is often delayed and empiric therapy is frequently inadequate.

### Limitations

While this study brings important data from summarizing previously published case reports on this rare entity it has notable limitations. First, publication bias is inevitable in this type of review, and we acknowledge this shortcoming. Second, the study sample is relatively small, and not all cases reported all variables of interest. Finally, some of the high-quality case reports might have been missed if they were not published in the journals indexed in the two databases we used, or if they were published in a language other than English or Portuguese.

## Conclusions

Patients with liver abscess due to aspergilosis have very high overall mortality, 38%, which is higher than in those with bacterial or amoebic etiology. These patients are often immunocompromised and diagnosis is delayed due to low sensitivity and specificity of diagnostic tests. Further prospective studies are urgently needed to evaluate novel biomarkers that might expedite diagnosis and improve the outcome in this patient population.

## Data Availability

All data generated or analysed during this study are included in this published article.
